# The Kansas City Hospital

**Published:** 1891-11

**Authors:** M. A. A. Wolff

**Affiliations:** 504–6 West Seventh Street, Kansas City, Mo.


### THE KANSAS CITY HOSPITAL,

504–6 West Seventh Street,

KANSAS CITY, MO., August 7th, 1891.

EDITOR HOMŒOPATHIC PHYSICIAN:

As I expect, you know I am yet an inmate of the Homœopathic Hospital, of Kansas City. The direction, however, given above, shows a change of locality. This change happened on July 31st, when all things belonging to the institution, as well as the patients, were removed to this new and grand abode, where there is room for hospital and college. It is a very imposing building, eighty feet front, one hundred and twenty-five feet depth. In the basement are kitchen, dining-room, and laundry. First floor is given to the college, lecture-rooms, clinical theatre, laboratory, etc., etc. On second floor the front parlors are the dwelling of the house physician, office, storeroom, and what is left (there are fourteen rooms on each floor) is, as well as the whole third floor, devoted to the hospital. The school may be proud of this grand new establishment, which is an ornament for the homœopathic profession. Let us wish it God-speed, as it deserves. M. A. A. WOLFF.

